# 

**Published:** 1897-09

**Authors:** 


### BOOKS RECEIVED.

Twentieth Century Practice. An International Encyclopedia of Modern Medical Science. By leading authorities of Europe and America. Edited by Thomas L. Stedman, M. D., New York City. In twenty volumes. Volume XI. Diseases of the Nervous System. New York: William Wood & Co. 1897.

The American Text-Book of Operative Dentistry. In contributions by eminent American authorities. Edited by Edward C. Kirk, D. D. S., Professor of Clinical Dentistry, University of Pennsylvania, Department of Dentistry. In one very handsome octavo volume of 699 pages, with 751 engravings. Cloth, $5.50 ; leather, $6.50 ; net. Philadelphia and New York : Lea Brothers & Co., Publishers.

Transactions of the Medical Society of the State of New York for the Year 1897. Frederic C. Curtis, M. D., Secretary. Philadelphia : William J. Dornan, Printer. 1897.

The Johns Hopkins Hospital Reports. Volume VI. Baltimore : The Johns Hopkins Press. 1897.

Annual Report of the State Board of Charities for the Year 1896. Transmitted to the legislature February 25, 1897. Albany and New York : Wynkoop Hallenbeck Crawford Co., State Printers. 1897.

The Menopause. A consideration of the phenomena which occur to women at the close of the child-bearing period, with incidental allusions to their relationship to menstruation. Also a particular consideration of the premature (especially the artificial) menopause. By Andrew F. Currier, A. B., M. D., New York City. Duodecimo, pp. xvi.—309. New York : D. Appleton. 1897.

The Roller Bandage with a Chapter on Surgical Dressing. By William Barton Hopkins, M. D., Surgeon to Pennsylvania Hospital. Illustrated. Fourth edition. Price, $1.25. Philadelphia : J. B. Lippincott Co. 1897.

International Clinics. A Quarterly of Clinical Lectures on Medicine, Neurology, Surgery, Gynecology, Obstetrics, Ophthalmology, Laryngology, Pharyngology, Rhinology, Otology and Dermatology, and specially prepared articles on treatment. By professors and lecturers in the leading medical colleges of the United States, Germany, Austria, France, Great Britain and Canada. Edited by Judson Daland, M. D., (Univ. of Penna.) Philadelphia, Instructor in Clinical Medicine and Lecturer on Physical Diagnosis in the University of Pennsylvania ; Assistant Physician to the Hospital of the University of Pennsylvania, etc.; Fellow of the College of Physicians of Philadelphia. J. Mitchell Bruce, M. D., F. R. C. P., London, England, Physician to, and Lecturer on, the Principles and Practice of Medicine at the Charing Cross Hospital. David W. Finlay, M. D., F. R. C. P., Aberdeen, Scotland, Professor of Practice of Medicine in the University of Aberdeen ; Physician to, and Lecturer on, Clinical Medicine in the Aberdeen Royal Infirmary, etc. Volume II. Seventh series. 1897. Octavo, pp. xii.—371. Philadelphia: J. B. Lippincott Co. 1897.

Annual of the Universal Medical Sciences and Analytical Index. A Yearly Report of the Progress of the General Sanitary Sciences Throughout the World. Edited by Charles E. Sajous, M. D., and seventy associate editors, assisted by over 200 corresponding editors, collaborators and correspondents. Illustrated with chromo-lithographs, engravings and maps. Five volumes. Philadelphia, New York, Chicago: The F. A. Davis Co., Publishers. London: F. J. Rebman. Australian agency: Melbourne, Victoria. 1897.

The Diseases of Women. A Handbook for Students and Practitioners. By J. Bland Sutton, F. R. C. S., Eng., Surgeon to the Chelsea Hospital for Women; Assistant Surgeon, Middlesex Hospital, London, and Arthur E. Giles, M. D., B. Sc., London, F. R. C. S., Edin., Assistant Surgeon, Chelsea Hospital for Women, London. Small 8vo, pp. 436. Illustrated. Price, $2.50, net. Philadelphia: W. B. Saunders, 925 Walnut street. 1897.

